# Association between angiotensin converting enzyme inhibitor or angiotensin receptor blocker use prior to major elective surgery and the risk of acute dialysis

**DOI:** 10.1186/1471-2369-15-53

**Published:** 2014-04-02

**Authors:** Mitesh Shah, Arsh K Jain, Steven M Brunelli, Steven G Coca, Philip J Devereaux, Matthew T James, Jin Luo, Amber O Molnar, Marko Mrkobrada, Neesh Pannu, Chirag R Parikh, Michael Paterson, Salimah Shariff, Ron Wald, Michael Walsh, Richard Whitlock, Duminda N Wijeysundera, Amit X Garg

**Affiliations:** 1 Divisions of Clinical Epidemiology and Internal Medicine, McGill University, Montreal, Canada; 2 Division of Nephrology, Department of Medicine, Western University, London, Canada; 3 Institute for Clinical Evaluative Sciences, Ontario, Canada; 4 Renal Division, Brigham and Women’s Hospital, Harvard Medical School, Boston, USA; 5 Section of Nephrology, Yale University School of Medicine, New Haven, USA; 6 Department of Clinical Epidemiology and Biostatistics, McMaster University, Hamilton, Canada; 7 Division of Cardiology, Department of Medicine, McMaster University, Hamilton, Canada; 8 Department of Medicine, University of Calgary, Hamilton, Canada; 9 Division of Nephrology, University of Ottawa, Ottawa, Canada; 10 Department of Medicine, Western University, London, Canada; 11 Division of Nephrology, Department of Medicine, University of Alberta, Edmonton, Canada; 12 Department of Family Medicine, McMaster University, Hamilton, Canada; 13 Division of Nephrology, University of Toronto, Toronto, Canada; 14 Division of Nephrology, Department of Medicine, McMaster University, Hamilton, Canada; 15 Department of Surgery, McMaster University, Hamilton, Canada; 16 Department of Anesthesia, University of Toronto, Toronto, Canada; 17 London Kidney Clinical Research Unit, Westminster, London Health Sciences Centre, Room ELL-101, 800 Commissioners Road East, London, Ontario N6A 4G5, Canada

**Keywords:** Acute dialysis, Angiotensin converting enzyme inhibitor, Angiotensin receptor blocker, Major elective surgery

## Abstract

**Background:**

Some studies but not others suggest angiotensin converting enzyme inhibitor (ACEi) or angiotensin receptor blocker (ARB) use prior to major surgery associates with a higher risk of postoperative acute kidney injury (AKI) and death.

**Methods:**

We conducted a large population-based retrospective cohort study of patients aged 66 years or older who received major elective surgery in 118 hospitals in Ontario, Canada from 1995 to 2010 (n = 237,208). We grouped the cohort into ACEi/ARB users (n = 101,494) and non-users (n = 135,714) according to whether the patient filled at least one prescription for an ACEi or ARB (or not) in the 120 days prior to surgery. Our study outcomes were acute kidney injury treated with dialysis (AKI-D) within 14 days of surgery and all-cause mortality within 90 days of surgery.

**Results:**

After adjusting for potential confounders, preoperative ACEi/ARB use versus non-use was associated with 17% lower risk of post-operative AKI-D (adjusted relative risk (RR): 0.83; 95% confidence interval (CI): 0.71 to 0.98) and 9% lower risk of all-cause mortality (adjusted RR: 0.91; 95% CI: 0.87 to 0.95). Propensity score matched analyses provided similar results. The association between ACEi/ARB and AKI-D was significantly modified by the presence of preoperative chronic kidney disease (CKD) (*P* value for interaction < 0.001) with the observed association evident only in patients with CKD (CKD - adjusted RR: 0.62; 95% CI: 0.50 to 0.78 versus No CKD: adjusted RR: 1.00; 95% CI: 0.81 to 1.24).

**Conclusions:**

In this cohort study, preoperative ACEi/ARB use versus non-use was associated with a lower risk of AKI-D, and the association was primarily evident in patients with CKD. Large, multi-centre randomized trials are needed to inform optimal ACEi/ARB use in the peri-operative setting.

## Background

Acute kidney injury (AKI) is a serious complication of major surgery and severe AKI requires treatment with acute dialysis [1,2]. Annually 230 million patients undergo major surgery worldwide and around one million major surgeries are complicated by AKI treated with dialysis (AKI-D) [1-4]. The development of AKI is associated with increased morbidity, mortality, and health care expenditures [5]. A patient’s risk for developing postoperative AKI is influenced by a variety of clinical factors including pre-existing comorbidities (e.g. chronic kidney disease (CKD), congestive heart failure, coronary artery disease, diabetes, hypertension), clinical factors around the time of surgery (e.g. volume depletion, blood loss, hypotension, administration of vasoconstrictors), type of surgery (e.g. cardiac, vascular), and certain medications used in the perioperative period (e.g. antihypertensive medications) [2,3,6-10]. Medication use prior to surgery is of particular interest given the possibility of modification prior to planned elective surgeries.

Angiotensin converting enzyme inhibitor (ACEi) and angiotensin receptor blocker (ARB) are frequently prescribed medications [11,12]. Despite their demonstrated benefits in outpatient settings, their continued use in the perioperative period remains controversial as preoperative ACEi/ARB use may lead to the development of perioperative hypotension and subsequent AKI [8,13-16]. However, previous observational studies on the association between preoperative ACEi/ARB use and AKI have had conflicting results, and have focused on milder forms of AKI rather than the most serious renal outcome of AKI-D. We conducted this large retrospective cohort study to test the hypothesis that preoperative ACEi/ARB use compared to non-use associates with a different risk of AKI-D following major elective surgery.

## Methods

### Setting and study overview

We conducted a retrospective, population-based cohort study of patients aged 66 years or older admitted to a hospital for a major elective surgery from 1995 to 2010 in Ontario, Canada. Ontario residents have universal access to hospital care and physician services and those aged 65 years or older have universal prescription drug coverage [17]. We performed this study according to a prespecified protocol approved by the research ethics board at Sunnybrook Health Sciences Centre, Toronto, Canada. The board deemed that patient informed consent was not required for this study which used datasets without patient identifiers. The reporting of this study follows guidelines set out for observational studies (detailed in Additional file 1) [18].

### Data sources

We used linked large health care databases including hospital discharge, physician billing, and prescription drug claims databases to determine patients’ characteristics, information on comorbidities, outcome data, and drug usage. The Canadian Institute for Health Information Discharge Abstract Database (CIHI-DAD) provides detailed diagnostic and procedural information on all hospitalizations. The Ontario Health Insurance Plan (OHIP) database records all health claims for inpatient and outpatient physician services. The Ontario Registered Persons Database (RPDB) records demographic and vital status information. The Ontario Drug Benefits (ODB) database provides highly accurate information on dispensed outpatient medications [17]. These databases have been used extensively to research health outcomes and health services, including drug-induced and perioperative AKI [3,19-23].

### Study population

We included patients aged 66 years or older admitted to a hospital for elective surgery between January 1, 1995 and November 30, 2010. We excluded patients in their first year of universal drug coverage (patients aged 65 years) to avoid incomplete historical medication data. We included five surgical categories: cardiac, vascular, thoracic, abdominal, and retro-peritoneal, which all carry an appreciable risk for AKI-D (sample database codes in Additional file 1) [2,3,6,7,22]. To avoid selecting emergent or urgent surgery, we excluded all surgeries done during the course of a non-surgical hospital admission. We also excluded hospital admissions associated with missing or invalid identification, or demographic information. To ensure all patients had a recent access to health-care services, we limited our analyses to patients with at least one dispensed outpatient medication prescription in the 120 days prior to surgery.

We excluded patients with end-stage renal disease (ESRD) prior to their surgery (i.e. any evidence of dialysis or kidney transplantation) as the assessment of AKI-D is different and may no longer be relevant. To avoid the need to account for less frequently prescribed anti-hypertensive medications in the analyses, we only considered patients with either no evidence of anti-hypertensive medication use, or evidence of a prescription for the following frequently prescribed anti-hypertensive medications in the 120 days prior to surgery: ACEi, ARB, β-blocker, calcium channel blocker, non-potassium sparing diuretic (loop diuretic or thiazide diuretic). For patients with multiple eligible surgeries during the study period, we randomly selected one surgical procedure to avoid within patient clustering in the analyses.

### Preoperative ACEi/ARB use

We grouped selected surgical cohort into ACEi/ARB users and non-users according to whether the patient filled at least one prescription for an ACEi or ARB (or not) in the 120 days prior to surgery. We selected a time frame of 120 days because the provincial drug plan requires each prescription to be renewed at least once every 100 days and 20 extra days were considered to accommodate any missed doses resulting in a longer period between renewal [24].

### Baseline characteristics

We assessed demographic characteristics and comorbidities using validated database codes whenever possible (detailed in Additional file 1) [25-32]. We also determined concomitant medication use in the 120 days prior to surgery.

### Primary and secondary outcomes

Our primary outcome was AKI-D within 14 days of surgery. AKI-D was determined using a set of high performance service fee codes (detailed in Additional file 1). Our secondary outcome was all-cause mortality within 90 days of surgery (in our data sources the code has a sensitivity of 97.8% and specificity of 100% for the finding of death) [33].

### Primary analyses

We performed all statistical analyses at the Institute for Clinical Evaluative Sciences using SAS 9.2 (SAS Institute, Cary, NC, USA). We assessed differences in baseline characteristics between ACEi/ARB users and non-users using standardized differences. As recommended by others, we considered a standardized difference of > 10% as a meaningful difference between the two groups [34]. To determine the association between ACEi/ARB use and study outcomes, we conducted multivariable logistic regression analyses and estimated adjusted odds ratios (ORs) and 95% confidence intervals (CIs). We interpreted odds ratios as relative risks (RRs) (appropriate given the incidences observed) [35]. In the logistic regression analyses, we adjusted for the following prespecified baseline characteristics associated with ACEi/ARB use and postoperative AKI [2,3,6-8,10,11,15,36,37]: age, sex, CKD, coronary artery disease, congestive heart failure, cerebrovascular disease, peripheral vascular disease, chronic obstructive pulmonary disease, chronic liver disease, type of surgery (cardiac, vascular, thoracic, abdominal, retro-peritoneal), era of surgery (1995 to 1998, 1999 to 2001, 2002 to 2004, 2005 to 2007, 2008 to 2010), and use of one or more drugs from the following medication groups - anti-diabetic medications, β-blockers, calcium channel blockers, non-potassium sparing diuretics, and statins. The presence of all of these characteristics was assessed with database codes (detailed in Additional file 1).

### Additional analyses

#### *Propensity score matching*

We derived a propensity score for every patient from all the potential confounders prespecified in our primary analysis. The propensity score indicated the likelihood of receiving a preoperative ACEi/ARB [38,39]. We derived a propensity score matched cohort of ACEi/ARB users and non-users, matching each ACEi/ARB user to a non-user on a one to one basis on the following characteristics: age (± 5 years), sex, CKD, and a caliper width of ± 0.2 standard deviation of the propensity score [3,38,39]. Each non-user could be selected once only. We assessed differences in baseline characteristics between matched cohort of ACEi/ARB users and non-users using standardized differences. To determine the association between ACEi/ARB use and study outcomes, we conducted conditional logistic regression analyses and estimated RRs and 95% CIs. We also calculated absolute risk reduction in study outcomes with ACEi/ARB use compared to non-use.

#### *Subgroup analyses*

CKD is a strong risk factor for postoperative AKI [7,10]. We performed sub-group analysis to explore whether pre-existing CKD was an effect modifier of the association between ACEi/ARB use and study outcomes. Additionally, we also performed sub-group analyses for congestive heart failure and diabetes (determined by anti-diabetic medications prescription). We adjusted for all potential confounders included in the primary analyses except the sub-group factor being tested. A *P* value < 0.05 for the test of interaction was considered statistically significant.

#### *Time to event analysis*

We performed multivariable Cox-proportional hazards regression analyses to examine the association between ACEi/ARB use and study outcomes accounting for the time to event. We adjusted for all prespecified potential confounders and estimated the adjusted hazard ratios (HRs) and 95% CIs.

## Results

### Baseline characteristics: ACEi/ARB users and non-users

We selected 237,208 adult patients from 118 hospitals in Ontario, of whom 101,494 (42.8%) were ACEi/ARB users (Table 1) (selection figure detailed in Additional file 1). ACEi/ARB users, compared to non-users, were more likely to be male and have CKD, cerebrovascular disease, peripheral vascular disease, coronary artery disease, and congestive heart failure. ACEi/ARB users, compared to non-users, were more likely to be on anti-diabetic medications, other anti-hypertensive medications (β-blockers, calcium channel blockers, and non-potassium sparing diuretics), and statins. ACEi/ARB users, compared to non-users, were more likely to undergo cardiac and vascular surgeries. Patients having their surgery in more recent years (2002 to 2010) were more likely to have received ACEi/ARB prior to surgery compared to prior years (1995 to 2001).

**Table 1:** Baseline characteristics: ACEi/ARB users and non-users

	**Pre-matched cohort**		**Propensity matched cohort**	
	**ACEi/ARB users**	**Non-users**	**ACEi/ARB users**	**Non-users**
	**N = 101,494**	**N = 135,714**	**N = 67,822**	**N = 67,822**
**Demographics**				
Women	41,034 (40.4%)	60,522 (44.6%)	29,425 (43.4%)	29,425 (43.4%)
Age at index date (years)	74 (70 to 78)	73 (69 to 78)	74 (70 to 78)	74 (70 to 78)
Age groups (years)				
66 to 70	30,557 (30.1%)	43,652 (32.2%)	20,098 (29.6%)	20,165 (29.7%)
71 to 75	30,966 (30.5%)	40,782 (30.0%)	20,482 (30.2%)	20,391 (30.1%)
76 to 80	23,898 (23.5%)	29,529 (21.8%)	15,982 (23.6%)	15,779 (23.3%)
81 to 85	11,812 (11.6%)	15,361 (11.3%)	8,101 (11.9%)	8,205 (12.1%)
86 to 90	3,651 (3.6%)	5,294 (3.9%)	2,682 (4.0%)	2,761 (4.1%)
91+	610 (0.6%)	1,096 (0.8%)	477 (0.7%)	521 (0.8%)
**Comorbidities**				
Chronic kidney disease	7,538 (7.4%)^a^	5,027 (3.7%)^a^	3,852 (5.7%)	3,852 (5.7%)
Coronary artery disease	67,921 (66.9%)^a^	61,137 (45.0%)^a^	40,114 (59.1%)	40,346 (59.5%)
Congestive heart failure	22,108 (21.8%)^a^	12,413 (9.1%)^a^	10,740 (15.8%)	10,317 (15.2%)
Cerebrovascular disease	18,016 (17.8%)^a^	17,551 (12.9%)^a^	10,970 (16.2%)	11,097 (16.4%)
Peripheral vascular disease	6,490 (6.4%)^a^	5,571 (4.1%)^a^	3,675 (5.4%)	3,599 (5.3%)
COPD	5,806 (5.7%)	7,121 (5.2%)	3,789 (5.6%)	3,527 (5.2%)
Chronic liver disease	295 (0.3%)	478 (0.4%)	200 (0.3%)	194 (0.3%)
**Medications**				
Oral hypoglycemic	21,267 (21.0%)^a^	10,381 (7.6%)^a^	8,766 (12.9%)	8,625 (12.7%)
Insulin	6,089 (6.0%)^a^	2,381 (1.8%)^a^	2,348 (3.5%)	1,937 (2.9%)
Anti-diabetic medication^b^	25,041 (24.7%)^a^	12,215 (9.0%)^a^	10,364 (15.3%)	10,076 (14.9%)
β-blocker	44,835 (44.2%)^a^	34,096 (25.1%)^a^	24,550 (36.2%)	24,702 (36.4%)
Calcium channel blocker	36,659 (36.1%)^a^	32,859 (24.2%)^a^	21,630 (31.9%)	22,131 (32.6%)
Diuretic^c^	40,345 (39.8%)^a^	22,042 (16.2%)^a^	19,830 (29.2%)	19,595 (28.9%)
Statin	53,915 (53.1%)^a^	31,691 (23.4%)^a^	27,348 (40.3%)	26,913 (39.7%)
**Surgical characteristics**				
**Type of surgery**				
Cardiac surgery	40,694 (40.1%)^a^	29,475 (21.7%)^a^	22,222 (32.8%)	22,380 (33.0%)
Vascular surgery	18,459 (18.2%)^a^	18,969 (14.0%)^a^	11,509 (17.0%)	11,904 (17.5%)
Thoracic surgery	5,771 (5.7%)	10,177 (7.5%)	4,561 (6.7%)	4,176 (6.2%)
Abdominal surgery	30,471 (30.0%)^a^	64,911 (47.8%)^a^	24,592 (36.2%)	24,630 (36.3%)
Retro-peritoneal surgery	6,099 (6.0%)^a^	12,182 (9.0%)^a^	4,938 (7.3%)	4,732 (7.0%)
**Era of surgery**				
1995 to 1998	14,718 (14.5%)^a^	45,173 (33.3%)^a^	13,278 (19.6%)	13,647 (20.1%)
1999 to 2001	16,900 (16.6%)^a^	30,122 (22.2%)^a^	13,457 (19.8%)	13,963 (20.6%)
2002 to 2004	20,960 (20.7%)^a^	21,992 (16.2%)^a^	13,404 (19.8%)	13,194 (19.5%)
2005 to 2007	23,711 (23.4%)^a^	19,520 (14.4%)^a^	13,625 (20.1%)	13,300 (19.6%)
2008 to 2010	25,205 (24.8%)^a^	18,907 (13.9%)^a^	14,058 (20.7%)	13,718 (20.2%)

ACEi: Angiotensin converting enzyme inhibitor; ARB: Angiotensin receptor blocker; COPD: Chronic obstructive pulmonary disease.

Data are presented as number (percent) except age at index date, which is presented as median (interquartile range). Index date is a surgical procedure date or hospital admission date (if the surgical procedure date is not available).

^a^Represents a standardized difference of >10% between ACEi or ARB users and non-users and we considered it as a meaningful difference [34]. Standardized difference is less sensitive to sample size compared to traditional hypothesis tests and is calculated by examining the difference between the two groups divided by the pooled standard deviation of the two groups [34].

^b^Anti-diabetic medication includes oral hypoglycemic and insulin. ^c^Diuretic include loop diuretic and thiazide diuretic. 3.0% (3,091/101,494) patients were on ACEi and ARB.

### Primary analyses: AKI-D and all-cause mortality

After adjusting for potential confounders, preoperative ACEi/ARB use was associated with a lower risk of AKI-D (adjusted RR: 0.83; 95% CI: 0.71 to 0.98) and a lower risk of all-cause mortality (adjusted RR: 0.91; 95% CI: 0.87 to 0.95) (Table 2; associations with all variables in the model are presented in Additional file 1).

**Table 2:** Association between preoperative ACEi/ARB use and outcomes

**Outcomes**	**No. of patients with events (percent)**		**Adjusted**^**a **^**RR (95% CI)**
	**ACEi/ARB users**	**Non-users**	
	**(N = 101,494)**	**(N = 135,714)**	
AKI-D	438 (0.43%)	372 (0.27%)	0.83 (0.71, 0.98)
All-cause mortality	4,654 (4.59%)	6,435 (4.74%)	0.91 (0.87, 0.95)

ACEi: Angiotensin converting enzyme inhibitor; ARB: Angiotensin receptor blocker; RR: Relative Risk; CI: Confidence Interval; AKI-D: Acute kidney injury treated with dialysis.

Outcomes: (1) Primary outcome: AKI-D (within 14 days of surgery); (2) Secondary outcome: All-cause mortality (within 90 days of surgery).

Relative risk was calculated for preoperative ACEi/ARB use compared to non-use.

^**a**^Adjusted for age, sex, chronic kidney disease, coronary artery disease, congestive heart failure, cerebrovascular disease, peripheral vascular disease, chronic obstructive pulmonary disease, chronic liver disease, anti-diabetic agents, beta-adrenergic blockers, calcium channel blockers, non-potassium sparing diuretics, statins, type of surgery (cardiac, vascular, thoracic, abdominal, retro-peritoneal), era of surgery (1995 to 1998, 1999 to 2001, 2002 to 2004, 2005 to 2007, 2008 to 2010).

### Additional analyses

#### *Propensity score matched analyses: AKI-D and all-cause mortality*

A total of 67,822 ACEi/ARB users were successfully matched to 67,822 non-users (Table 1). Baseline characteristics were well balanced between matched ACEi/ARB users and non-users (Table 1). Similar to the primary analyses, preoperative ACEi/ARB use was associated with a lower risk of AKI-D (RR: 0.77; 95% CI: 0.65 to 0.92) and a lower risk of all-cause mortality (RR: 0.93; 95% CI: 0.88 to 0.97) (Table 3). ACEi/ARB use, compared to non-use, was associated with 0.09% (95% CI: 0.03% to 0.16%) absolute risk reduction in AKI-D and 0.35% (95% CI: 0.12% to 0.57%) absolute risk reduction in all-cause mortality.

**Table 3:** Propensity score matched cohort: association between preoperative ACEi/ARB use and outcomes

**Outcomes**	**No. of patients with events (percent)**		**RR (95% CI)**	**ARR (95% CI)**
	**ACEi/ARB users**	**Non-users**		
	**(N = 67,822)**	**(N = 67,822)**		
AKI-D	215 (0.32%)	278 (0.41%)	0.77 (0.65, 0.92)	0.09% (0.03%, 0.16%)
All-cause mortality	3,060 (4.51%)	3,295 (4.86%)	0.93 (0.88, 0.97)	0.35% (0.12%, 0.57%)

ACEi: Angiotensin converting enzyme inhibitor; ARB: Angiotensin receptor blocker; RR: Relative Risk; CI: Confidence Interval; ARR: Absolute risk reduction; AKI-D: Acute kidney injury treated with dialysis.

Outcomes: (1) Primary outcome: AKI-D (within 14 days after surgery); (2) Secondary outcome: All-cause mortality (within 90 days after surgery).

Relative risk was calculated for preoperative ACEi or ARB use compared to non-use.

#### *Subgroup analyses: AKI-D and all-cause mortality*

We found CKD significantly modified the association between ACEi/ARB use and AKI-D (test of interaction, *P* value < 0.001). The observed benefit was evident in patients with CKD (CKD - adjusted RR: 0.62; 95% CI: 0.50 to 0.78 and not in those without CKD: adjusted RR: 1.00; 95% CI: 0.81 to 1.24) (Table 4). There was no significant interaction between ACEi/ARB use and CKD for all-cause mortality (test of interaction, *P* value = 0.26). We did not find a significant interaction between ACEi/ARB use and the outcomes in subgroups of patients defined by the presence of congestive heart failure and diabetes (Table 4).

**Table 4:** Influence of chronic kidney disease, congestive heart failure, and diabetes on association between preoperative ACEi/ARB use and outcomes

**Outcomes**	**Presence/absence of comorbid condition**		**Adjusted**^**a **^**RR (95% CI)**	***P *** **value (test for interaction)**
AKI-D	CKD	Yes	0.62 (0.50, 0.78)	< 0.001
		No	1.00 (0.81, 1.24)	
	CHF	Yes	0.62 (0.45, 0.85)	0.29
		No	0.81 (0.65, 1.00)	
	Diabetes	Yes	0.63 (0.43, 0.91)	0.56
		No	0.77 (0.63, 0.94)	
All-cause mortality	CKD	Yes	0.86 (0.75, 0.98)	0.26
		No	0.91 (0.87, 0.96)	
	CHF	Yes	1.01 (0.91, 1.12)	0.06
		No	0.92 (0.86, 0.97)	
	Diabetes	Yes	0.96 (0.86, 1.09)	0.34
		No	0.93 (0.88, 0.99)	

ACEi: Angiotensin converting enzyme inhibitor; ARB: Angiotensin receptor blocker; RR: Relative risk; CI: Confidence interval; AKI-D: Acute kidney injury treated with dialysis; CKD: Chronic kidney disease; CHF: Congestive heart failure.

Outcomes: (1) Primary outcome: AKI-D (within 14 days after surgery); (2) Secondary outcome: All-cause mortality (within 90 days after surgery).

Relative risk was calculated for preoperative ACEi or ARB use compared to non-use.

^a^CKD sub-group analyses: adjusted for age, sex, coronary artery disease, congestive heart failure, cerebrovascular disease, peripheral vascular disease, chronic obstructive pulmonary disease, chronic liver disease, anti-diabetic agents, beta-adrenergic blockers, calcium channel blockers, non-potassium sparing diuretics, statins, type of surgery (cardiac, vascular, thoracic, abdominal, retro-peritoneal), era of surgery (1995 to 1998, 1999 to 2001, 2002 to 2004, 2005 to 2007, 2008 to 2010).

^a^CHF sub-group analyses: adjusted for age, sex, coronary artery disease, chronic kidney disease, cerebrovascular disease, peripheral vascular disease, chronic obstructive pulmonary disease, chronic liver disease, anti-diabetic agents, beta-adrenergic blockers, calcium channel blockers, non-potassium sparing diuretics, statins, type of surgery (cardiac, vascular, thoracic, abdominal, retro-peritoneal), era of surgery (1995 to 1998, 1999 to 2001, 2002 to 2004, 2005 to 2007, 2008 to 2010).

^a^Diabetes sub-group analyses: adjusted for age, sex, coronary artery disease, chronic kidney disease, congestive heart failure, cerebrovascular disease, peripheral vascular disease, chronic obstructive pulmonary disease, chronic liver disease, beta-adrenergic blockers, calcium channel blockers, non-potassium sparing diuretics, statins, type of surgery (cardiac, vascular, thoracic, abdominal, retro-peritoneal), era of surgery (1995 to 1998, 1999 to 2001, 2002 to 2004, 2005 to 2007, 2008 to 2010).

#### *Time to event analyses: AKI-D and all-cause mortality*

Similar to the primary analyses, preoperative ACEi/ARB use was associated with a lower risk of AKI-D (adjusted HR: 0.83; 95% CI: 0.71 to 0.97) and all-cause mortality (adjusted HR: 0.91; 95% CI: 0.88 to 0.95).

## Discussion

Contrary to the results of other studies, in this analysis preoperative ACEi/ARB use versus non-use was associated with 17% lower risk of AKI-D and 9% lower risk of all-cause mortality, when we adjusted for a set of prespecified potential confounders. We found similar results in the propensity score matched analyses. The association of preoperative ACEi/ARB use versus non-use on AKI-D was only evident in patients with CKD.

With respect to mechanism, it is possible that potential benefits of ACEi/ARB use in surgical settings relates to angiotensin II inhibition [36,40-42]. Angiotensin II is a potent vasoconstrictor, which can cause postoperative AKI (i.e., true tubular injury) by increasing oxidative stress, endothelial dysfunction, inflammatory response, and renal vascular resistance, and by reducing renal blood flow [36,42]. By inhibiting angiotensin II, ACEi/ARB use may increase the risk of functional AKI (i.e., a drop in GFR) but may paradoxically reduce the risk of “true AKI” (true tubular injury) [36,40-42]. In this regard, ACEi and ARB may improve renal blood flow and oxygenation to the renal tubules by vasodilatation of efferent arterioles and may prevent tubular cell necrosis during ischemic insults around surgery [13]. To wit, a recent analysis from a large multi-center cohort of patients undergoing cardiac surgery (TRIBE-AKI cohort) showed that while AKI defined by changes in serum creatinine, was higher in those that had ACEi or ARB continued pre-operatively, there was not a concomitant increase in several biomarkers of kidney injury [43]. While that study may also suffer confounding by indication, most impressive is that despite greater comorbidities in patients that continued ACEi/ARB, the kidney injury biomarkers still were not more elevated, suggesting some protective benefit of these agents [43].

Our results are also similar to two small observational studies by Benedetto *et al*. and Barodka *et al*. [40,41] Benedetto *et al*. studied 536 patients who underwent cardiac surgery and observed a lower risk of AKI with preoperative ACEi use compared to non-use (adjusted OR: 0.48; 95% CI: 0.23 to 0.77) [41]. The authors presumed the pathophysiologic benefit of ACEi use stemmed from the preservation of renal blood flow during surgery [41]. Barodka *et al*. found similar benefits with preoperative ACEi/ARB use compared to non-use in 346 patients who underwent cardiac surgery (adjusted OR: 0.19; 95% CI: 0.04 to 0.84) [40].

Other observational studies by Rady *et al*. [44], Ouzounian *et al.*[37], and Yoo *et al.*[45], studied 11330, 1647, and 472 cardiac surgery patients, respectively, and demonstrated no significant association between preoperative ACEi/ARB use and AKI. However, all three studies observed a non-significant trend towards a benefit with preoperative ACEi/ARB use raising the possibility of insufficient statistical power to detect the association [37,44,45].

Miceli *et al*. performed a propensity score matched analysis in 9,274 patients who underwent cardiac surgery and noted a 1.36 fold higher risk of AKI (adjusted OR: 1.36; 95% CI: 1.10 to 1.67) with preoperative ACEi/ARB use compared to non-use and a two-fold higher risk of mortality (adjusted OR: 2.00; 95% CI: 1.17 to 3.42) [8]. The authors speculated that the AKI occurred as a result of a decrease in renal perfusion, mainly due to reduction in mean arterial pressure along with increased use of vasoconstrictors [8]. Both Cittanova *et al.* and Arora *et al*. also observed an increased risk of AKI with preoperative ACEi and ARB use, although the study samples were small (249 aortic surgery patients and 1358 cardiac surgery patients, respectively) [11,46]. Railton *et al*. studied the outcome of AKI-D and observed a higher incidence of AKI-D with ACEi/ARB use compared to non-use in patients who underwent abdominal aortic aneurysm repair (4.6% vs. 0.8%; *P* value = 0.01) [47]. However, the number of patients (n = 883) and number of events (n = 24) were small [47].

Major considerations when comparing our results with previous studies are the heterogeneous AKI definitions, type of surgery, and the consideration of preoperative CKD in statistical analysis. Our primary outcome was AKI-D, which is the acute renal outcome most important to patients and their health-care providers [1-3,5]. The outcome of AKI-D is distinct from AKI defined solely by acute changes in serum creatinine [48]. The latter is a surrogate outcome and may be misleading particularly in the ACEi or ARB setting (i.e. use of these medications in outpatient settings may increase serum creatinine concentration despite evidence that the drugs prevent progression to ESRD requiring chronic maintenance dialysis) [11,49,50].

CKD is considered to be the most important risk factor for AKI [7,10]. However, the majority of previous studies did not account for CKD in their analyses [8,11,37,40,41,44-46]. In this study, we observed an association of less AKI-D with preoperative ACEi/ARB use compared to non-use only in patients with preoperative CKD . One concern interpreting this result is that CKD patients not receiving preoperative ACEi/ARB may represent an advanced disease population where ACEi/ARB might have been stopped due to the risk of early onset chronic maintenance dialysis [51]. Another concern is that we identified CKD patients using database codes which have limited validity. These codes underestimate CKD prevalence may have impacted the study results. Moreover, this prevented us from examining CKD stages according to preferred glomerular filteration rate (eGFR) categories [30,52].

### Study strengths and limitations

Our study has several strengths. To the best of our knowledge, this is the largest study to describe the association between preoperative ACEi/ARB use and AKI-D (over 230,000 patients from 118 hospitals). We included both cardiac and non-cardiac major surgeries. Unlike other studies, the large number of events of AKI-D (810 events) reduced concerns about statistical overfitting [53]. Given there is <1% yearly emigration from Ontario, the loss to follow up was minimal. Finally, the information available within the large Ontario health care databases reflects routine clinical practice and may be less prone to participation biases that can arise in other types of studies [3,19-23].

There are several limitations to our study. Due to possible difference in underlying mechanism for the risk of postoperative acute dialysis in emergent surgeries compared to elective surgeries, we excluded emergent surgeries from our analyses. In an emergency situation it is also difficult to manipulate pre-operative medication use. However, exclusion of emergency surgeries may have reduced the generalizability of the study results. We could not determine medication compliance from evidence of a dispensed prescription for ACEi, ARB, or other medications in our data sources. Our study was limited by the absence of key information on perioperative ACEi/ARB use particularly whether it was held prior to surgery and if so when it was held, and when it was restarted after surgery. Important information such as preoperative and intraoperative blood pressure was also not available. These are key elements to guide the optimal and safe use of ACEi/ARB use in the perioperative period, including regimens to be tested in large randomized controlled trials (RCTs). We were also not able to adjust for variables unavailable in our data sources such as body mass index, preoperative proteinuria, non-prescription medication use and in hospital medication use. Another limitation with our data sources is the accuracy of codes for patient related health information. In attempt to limit these concerns, we did use database codes supported by validation studies whenever possible [25-32].

### Study implications and future directions

Our study results support the need for RCTs in this setting, to test a regimen of perioperative ACEi/ARB selected for optimal efficacy and safety. Given the low incidence of acute dialysis (about 0.45% in all types of major surgery [1-3]), a very large trial (over 150,000 patients) would be needed to examine a meaningful difference with this as a primary outcome. Such a trial is unlikely to occur. However, if we consider a primary outcome of 90-day all-cause mortality (4.67% of patients in our study), the sample size is tenable at about 15,000 patients. The sample size could be further reduced if there is a rationale to consider a composite of clinically important events including peri-operative cardiac events. Enrolling a large number of patients with CKD may be prudent, as in the current study the signal of benefit for AKI-D was strongest in this group of patients. Given the large number of surgeries complicated by AKI-D worldwide each year, we propose that such RCTs should be undertaken.

## Conclusions

In this cohort study, preoperative ACEi/ARB use versus non-use was associated with a lower risk of AKI-D, and the association was primarily evident in patients with CKD. Large, multi-centre randomized trials are needed to inform optimal ACEi/ARB use in the peri-operative setting.

## Competing interests

Amit Garg’s institution received an unrestricted grant from Pfizer for research unrelated to the current project. All the authors declare no competing interests.

## Authors’ contributions

MS, AJ and AG conceived of the study and developed the study protocol. MS, AJ, SB, SC, PD, MJ, JL, AM, MM, NP, CP, MP, SS, RW, MW, RW, DW and AG contributed to the study design. JL carried out the statistical analysis. MS drafted the manuscript. MS, AJ, SB, SC, PD, MJ, JL, AM, MM, NP, CP, MP, SS, RW, MW, RW, DW and AG read, edited and approved the final manuscript.

## Pre-publication history

The pre-publication history for this paper can be accessed here:

http://www.biomedcentral.com/1471-2369/15/53/prepub

## Supplementary Material

Additional file 1 Additional details on research methods and results. Click here for file
