# Supplementary material for: Association between angiotensin converting enzyme inhibitor or angiotensin receptor blocker use prior to major elective surgery and the risk of acute dialysis
Source: BMC Nephrol. 2014 Apr 2;15:53. doi: 10.1186/1471-2369-15-53 (PMC4021413; doi:10.1186/1471-2369-15-53)
Supplement: Additional file 1 — Additional details on research methods and results. [file 1471-2369-15-53-S1.doc]

**SUBJECTS AND METHODS**

| Appendix A: STROBE checklist^1^ | | | |
| --- | --- | --- | --- |
|  | Item No | Recommendation | Location |
| **Title and abstract** | 1 | (*a*) Indicate the study’s design with a commonly used term in the title or the abstract | Abstract |
|  |  | (*b*) Provide in the abstract an informative and balanced summary of what was done and what was found | Abstract |
| Introduction | | |  |
| Background/  rationale | 2 | Explain the scientific background and rationale for the investigation being reported | Introduction |
| Objectives | 3 | State specific objectives, including any pre-specified hypotheses | Introduction |
| Methods | | |  |
| Study design | 4 | Present key elements of study design early in the paper | Methods |
| Setting | 5 | Describe the setting, locations, and relevant dates, including periods of recruitment, exposure, follow-up, and data collection | Methods |
| Participants | 6 | (*a*) Give the eligibility criteria, and the sources and methods of selection of participants. Describe methods of follow-up | Methods |
|  |  | (*b*) For matched studies, give matching criteria and number of exposed and unexposed | Methods, Table 1 |
| Variables | 7 | Clearly define all outcomes, exposures, predictors, potential confounders, and effect modifiers. Give diagnostic criteria, if applicable | Methods, Appendix |
| Data sources/ measurement | 8 | For each variable of interest, give sources of data and details of methods of assessment (measurement). Describe comparability of assessment methods if there is more than one group | Methods, Appendix |
| Bias | 9 | Describe any efforts to address potential sources of bias | Methods |
| Study size | 10 | Explain how the study size was arrived at | Using existing records |
| Quantitative variables | 11 | Explain how quantitative variables were handled in the analyses. If applicable, describe which groupings were chosen and why | Methods |
| Statistical methods | 12 | (*a*) Describe all statistical methods, including those used to control for confounding | Methods |
|  |  | (*b*) Describe any methods used to examine subgroups and interactions | Methods |
|  |  | © Explain how missing data were addressed | Data was complete |
|  |  | (*d*) If applicable, explain how loss to follow-up was addressed | Follow-up was complete |
|  |  | (*e*) Describe any sensitivity analyses | See results – additional analyses |
| Results | | |  |
| Participants | 13 | (a) Report numbers of individuals at each stage of study—eg numbers potentially eligible, examined for eligibility, confirmed eligible, included in the study, completing follow-up, and analysed | Appendix E |
|  |  | (b) Give reasons for non-participation at each stage | Appendix E |
|  |  | (c) Consider use of a flow diagram | Appendix E |
| Descriptive data | 14 | (a) Give characteristics of study participants (eg demographic, clinical, social) and information on exposures and potential confounders | Table 1 |
|  |  | (b) Indicate number of participants with missing data for each variable of interest | n/a |
|  |  | (c) Summarise follow-up time (eg, average and total amount) | n/a |
| Outcome data | 15 | Report numbers of outcome events or summary measures over time | Results,Table 2, Table 3 |
| Main results | 16 | (*a*) Give unadjusted estimates and, if applicable, confounder-adjusted estimates and their precision (eg, 95% confidence interval). Make clear which confounders were adjusted for and why they were included | Table 2 |
|  |  | (*b*) Report category boundaries when continuous variables were categorized | Table 1 |
|  |  | (*c*) If relevant, consider translating estimates of relative risk into absolute risk for a meaningful time period | n/a |
| Other analyses | 17 | Report other analyses done—eg analyses of subgroups and interactions, and sensitivity analyses | Results |
| Discussion | | |  |
| Key results | 18 | Summarise key results with reference to study objectives | Discussion |
| Limitations | 19 | Discuss limitations of the study, taking into account sources of potential bias or imprecision. Discuss both direction and magnitude of any potential bias | Discussion |
| Interpretation | 20 | Give a cautious overall interpretation of results considering objectives, limitations, multiplicity of analyses, results from similar studies, and other relevant evidence | Discussion |
| Generalisability | 21 | Discuss the generalisability (external validity) of the study results | Discussion |
| Other information | | |  |
| Funding | 22 | Give the source of funding and the role of the funders for the present study and, if applicable, for the original study on which the present article is based | Acknowledgements |

**SUBJECTS AND METHODS**

**Appendix B**: Sample of codes used to identify surgical procedures

| **SURGERY CLASS** | **DATABASE** | **SAMPLE OF CODES USED** |
| --- | --- | --- |
| Cardiac | CIHI- procedure | CCI: 1IJ76 (Bypass, coronary arteries),  1HU80 (Repair, mitral valve) |
|  | CIHI- procedure | CCP: 47.22 (Replacement of mitral valve with tissue graft), 48.09 (Other removal of coronary artery obstruction) |
| Thoracic | CIHI- procedure | CCI: 1GV87 (Excision partial, pleura), 1GR91 (Excision radical, lobe of lung), |
|  | CIHI- procedure | CCP: 54.0 (Esophagotomy), 44.5 (Complete pneumonectomy) |
| Vascular | CIHI- procedure | CCI: 1KE76 (Bypass, abdominal arteries), 1JK57 (Extraction, subclavian artery) |
|  | CIHI- procedure | CCP: 50.14 (Endarterectomy of aorta), 50.34 (Resection of aorta with replacement) |
| Abdominal | CIHI- procedure | CCI: 1NM80LA (Repair, large intestine open approach using apposition technique), 1NF87RK (Excision partial, stomach with vagotomy open approach gastrojejunal anastomosis) |
|  | CIHI- procedure | CCP: 53.51 (Excision of accessory spleen), 57.55 (Left hemicolectomy) |
| Retro-peritoneal | CIHI- procedure | CCI: 1PG80 (Repair, ureter),  1PB87 (Excision, partial, adrenal gland) |
|  | CIHI- procedure | CCP: 69.4 (Partial cystectomy), 20.2 (Bilateral adrenalectomy) |

CCI: The Canadian Classification of Health Interventions; CCP: The Canadian Classification of Diagnostic, Therapeutic and Surgical Procedures; CIHI-DAD: Canadian Institute for Health Information Discharge Abstract Database;

Juurlink *et al*.^2^ performed a CIHI-DAD validation study and observed a high sensitivity 0.95 (interquartile range (IQR): 0.89 to 0.99), and positive predictive value 0.91 (IQR: 0.82 to 0.97) to identify surgical procedures using CCI codes. The CIHI-DAD considers CCP prior to 2002 and CCI thereafter to identify surgical procedures.

**SUBJECTS AND METHODS**

**Appendix C**: Database codes used to identify comorbidities

| **Exclusion: Dialysis and kidney transplantation codes** | | | |
| --- | --- | --- | --- |
|  |  | **Codes** | **Validation** |
| 1. | Dialysis | ICD-9: “V45.1”, “V56.0”, “V56.8” | V45.1, V56.0, V56.8: Sensitivity^3^ = 90.4%; Specificity^3^ = 93.8%;  PPV^3^ = 94.0%;  NPV^3^ = 90.0% |
|  |  | ICD10: "T824", "Y602", "Y612", "Y622", "Y841", "Z49", "Z992", "N180", "E1022", "E1023", "E1122", "E1123", "E1322", "E1323", "E1422", "E1423" |  |
|  |  | CCP: “51.27”, “51.42”, “51.43”, “51.95”, “66.98” |  |
|  |  | CCI: "1OT53DATS", "1OT53HATS", "1OT53LATS", "1PZ21", "1SY55LAFT", "7SC59QD", "1KY76" |  |
|  |  | OHIP FEE: "R850", "G324", "G336", "G327", "G862", "G865", "G099", "R825", "R826", "R827", "R833", "R840", "R841", "R843", "R848", "R851", "Z450", "Z451", "Z452", "G864", "R852", "R853", "R854", "R885", "G333", "H540", "H740" |  |
|  |  |  |  |
| 2. | Kidney Transplantation | ICD-9: “V42” |  |
|  |  | ICD-10: “N165”, “Z940”, “T86100”, “T86101”, “T86102” |  |
|  |  | CCP: “67.43”, “67.5” |  |
|  |  | CCI: “1PC85LAXXJ”, "1PC85LAXXK" |  |
|  |  | OHIP FEE: “E762”, “S435”, “E769”, “S434”, “E771”, “Z631”, “G347”, “G348”, “G412”, “G408”, “G409” |  |

| **Comorbidities** | | | |
| --- | --- | --- | --- |
|  |  | **Codes** | **Validation** |
| 1. | Chronic kidney disease (CKD) | ICD-9: “403.0”, “403.1”, “403.9”, “404.0”, “404.1”, “404.9”, “582”, “583”, “580”, “581”, “584”, “585”, “586”, “587”, “588.0”, “588.8”, “588.9”, “593.7” | 583 to 586 (ICD-9) and N00, N04, N08, N18, N19 (ICD-10):  Sensitivity^4^ = 28.3%;  Specificity^4^ = 94.6%;  PPV^4^ = 51.9%;  NPV^4^ = 86.5%;  (Case definition for CKD: 1 claim or 1 hospitalization in past 3 years; compared with the reference standard of eGFR < 60 mL/min/1.73m^2^) |
|  |  | ICD-10: “I12”, “I13”, “N01”, “N03”, “N05”, “N07”, “N14”, “N15”, “N00”, “N04”, “N08”, “N18”, “N19”, “N26”, “N25”, “N137”, “N280”, “N2888”, “N06”, “N391” |  |
|  |  | OHIP DX: “403”, “580”, “581”, “585” |  |
|  |  |  |  |
| 2. | Chronic obstructive pulmonary disease | ICD-9: "491", "492", "496" | Sensitivity^5^ = 85.0%; Specificity^5^ = 78.4%  (Validated for patients > 35 years) |
|  |  | ICD-10: "J41", "J42", "J43", "J44" |  |
|  |  |  |  |
| 3. | Cerebrovascular disease | ICD-9: "430", "432.1", "433", "435", “436”, "437", "438", "362.3" | 436: PPV^6^ = 78% |
|  |  | ICD-10: "I60", "I61", "I63", "I64", "I65", "I66", "I67", "I68", "I69", "G45", "G46", "H34" | I60, I61, I63 to I69, G45,G46:  Sensitivity^7^ = 89%; PPV^7^ = 93% |
|  |  | CCP: "50.11", "50.12", "51.28" |  |
|  |  | CCI: "1JE50", "1JE57", "1JE87", "1JW50", "1JX57", "1JW57", "1JW76" |  |
|  |  | OHIP FEE: "R792", "N220", "N223", "J050", "N104", "N157", "N120" |  |
|  |  | OHIP DX: "432", "435", "436", "437" |  |
|  |  |  |  |
| 4. | Peripheral vascular disease | ICD-9: "440.0", "440.2", "440.8", "440.9", "557.1", "443.9", "444" |  |
|  |  | ICD-10: "I700", "I702", "I708", "I709", "I731", "I738", "I739", "K551" | I739: Sensitivity^7^ = 74%; PPV^7^ = 62%;  I700, I702, I708, I709, I731, I738:  PPV^8^ = 100% |
|  |  | CCP: "51.25", "51.29", "50.14", "50.16", "50.18", "50.28", "50.38" |  |
|  |  | CCI: "1KG76MI", "1KA76", "1KA50", "1KE76", "1KG26", "1KG50", "1KG57", "1KG87" |  |
|  |  | OHIP FEE: "R787", "R780", "R797", "R804", "R809", "R875", "R815", "R936", "R783", "R784", "R785", "E626", "R814", "R786", "R937", "R860", "R861", "R855", "R856", "R933", "R934", "R791", "R794", "E672", "R813", "R867", "E649" |  |
|  |  |  |  |
| 5. | Coronary artery disease | ICD-9: "412", "414", "429.2", "429.5", "429.6", "429.7" |  |
|  |  | ICD-10: "I20", "I21", "I22", "I23", "I24", "I25", "Z955", "Z958", "Z959", "R931", "T822" | I21, I22, I25:  Sensitivity^7^ = 86%, PPV^7^ = 96%;  I20: Sensitivity^2^ = 82%; PPV^2^ = 52%;  I23: PPV^8^ = 98% |
|  |  | CCP: "48.01", "48.02", "48.03", "48.04", "48.05", "48.1", "48.2", "48.3" |  |
|  |  | CCI: "1IJ26", "1IJ27", "1IJ50", "1IJ54", "1IJ57", "1IJ76" |  |
|  |  | OHIP FEE: "R741", "R742", "R743", "G298", "E646", "E651", "E652", "E654", "E655", "G262", "Z434", "Z448" |  |
|  |  | OHIP DX: "410", "412", "413" | 410:  Sensitivity^9^ = 88.8%;  Specificity^9^ = 92.8%;  PPV^9^ = 88.5%  413:  Sensitivity^9^ = 57.9%; Specificity^9^ = 93.9%;  PPV^9^ = 78.1% |
|  |  |  |  |
| 6. | Congestive heart failure | ICD-9: "425", "518.4", "514" |  |
|  |  | ICD-10: "I255", "I500", "I501", "I509", "J81" | I500, I501:  Sensitivity^7^ = 86%;  PPV^7^ = 86%; |
|  |  | CCP: "49.61", "49.62" |  |
|  |  | CCI: "1HP53" |  |
|  |  | OHIP FEE: "R701", "R702" |  |
|  |  | OHIP DX: "428" | 428:  Sensitivity^9^ = 58.5%; Specificity^9^ = 96.8%;  PPV^9^ = 65.1% |
|  |  |  |  |
| 7. | Chronic liver disease | ICD-9: "571.0", "571.1", "571.2", "571.3", "571.5", "571.6", "070.2", "070.3", "070.4", "070.5", "V02.6" | PPV^10^ = 43 to 93%;  NPV^10^ = 77 to 100%; |
|  |  | ICD-10: "K73", "K702", "K703", "K717", "K740","K742", "K743", "K744", "K745", "K746", "K721", "K729", "K766", "K767" | K73, K702, K703, K717, K740, K742, K743, K744, K745, K746:  Sensitivity^7^ = 58%;  PPV^7^ = 69%;  K721, K729, K766, K767:  Sensitivity^7^ = 86%;  PPV^7^ = 63%; |

ICD-9: International classification of disease 9^th^ version; ICD-10: International classification of disease 10^th^ version; CCI: The Canadian Classification of Health Interventions; CCP: The Canadian Classification of Diagnostic, Therapeutic and Surgical Procedures; OHIP FEE – Ontario health insurance plan fee codes; OHIP DX: Ontario health insurance plan diagnostic codes;

**SUBJECTS AND METHODS**

**Appendix D:** OHIP fee codes for the outcome of acute dialysis

| R849 | Dialysis - haemodialysis - initial & acute |
| --- | --- |
| G323 | Dialysis - haemodialysis - acute, repeat (first 3 services) |
| G866 | Intermittent haemodialysis treatment centre |
| G330 | Peritoneal dialysis - acute (up to 48 hours) |
| G331 | Peritoneal dialysis - repeat acute (up to 48 hours) (first 3 services) |
| G093 | Haemodiafiltration - continuous - initial & acute (first 3 services) |
| G095 | Slow continuous ultrafiltration - initial & acute (first 3 services) |
| G294 | Arteriovenous slow continuous ultrafiltration - initial and acute  (first 3 services) |
| G295 | Continuous arteriovenous haemofiltration - initial and acute  (first 3 services) |

OHIP fee codes – Ontario health insurance plan fee codes

**RESULTS**

**Appendix E:** Flow diagram: Patient selection

Hospital admissions for following types of surgery: cardiac, vascular, thoracic, abdominal, and retro-peritoneal during 1995 to 2010 in 118 hospitals in Ontario, Canada

Total No. of hospital admissions: 1,762,271 (100%)

.

Excluded 1,486,482 (84.35%) hospital admissions with:

- Surgery performed during non-surgical hospital admissions: 915,524 (51.95%)
- Coding error for acute dialysis at one of the hospitals: 7,145 (0.41%)
- Death prior to index date: 172 (0.01%)
- Missing or invalid identification or demographic information (age and sex): 19,934 (1.13%)
- Patients < 66 years: 487,049 (27.64%)
- Non-Ontario residents: 80 (~0.00%)
- Past history of dialysis or renal transplantation in 3 years prior to surgery: 7,180 (0.41%)
- No evidence of Ontario Drug Benefit plan use in the past 120 days prior to surgery: 14,315 (0.81%)
- Patients on rarely used anti-hypertensive medications in Ontario: 35,083 (1.99%)

Hospital admissions (for elective surgery) meeting inclusion criteria: 275,789

Randomly selected one surgical procedure for patients with multiple eligible surgical procedures and excluded 38,581 (2.19%) hospital admissions

Hospital admission for patients with one elective surgical procedure: 237,208

**RESULTS**

**RESULTS**

**Appendix F (Association between preoperative ACEi/ARB use and outcomes) showing all the variables considered in the logistic regression analysis**

| **Outcome: AKI-D** |  |
| --- | --- |
| **Variables** | **RR (95% CI)** |
|  |  |
| ACEi or ARB (Yes vs No) | 0.83 (0.71, 0.98) |
| Age | 0.99 (0.98, 1.01) |
| Sex (Female vs Male) | 0.88 (0.76, 1.02) |
| Chronic obstructive pulmonary disease | 1.44 (1.14, 1.81) |
| Cerebrovascular disease | 0.89 (0.74, 1.07) |
| Peripheral vascular disease | 0.69 (0.52, 0.91) |
| Coronary artery disease | 0.86 (0.72, 1.03) |
| Congestive heart failure | 1.36 (1.14, 1.61) |
| Chronic kidney disease | 11.04 (9.49, 12.86) |
| Chronic lung disease | 0.99 (0.36, 2.67) |
| Antidiabetic medication | 1.39 (1.18, 1.63) |
| β-blocker | 1.07 (0.92, 1.25) |
| Calcium channel blocker | 1.66 (1.44, 1.92) |
| Diuretic | 1.63 (1.39, 1.90) |
| Statin | 0.81 (0.69, 0.95) |
| Surgical Category |  |
| Cardiac vs Abdominal | 2.37 (1.94, 2.90) |
| Retroperitoneal vs Abdominal | 0.39 (0.24, 0.66) |
| Thoracic vs Abdominal | 0.89 (0.59, 1.36) |
| Vascular vs Abdominal | 2.36 (1.90, 2.92) |
| Era of surgery |  |
| 1999-2001 vs 1995-1998 | 0.81 (0.64, 1.02) |
| 2002-2004 vs 1995-1998 | 0.98 (0.78, 1.22) |
| 2005-2007 vs 1995-1998 | 0.88 (0.70, 1.11) |
| 2008-2010 vs 1995-1998 | 0.93 (0.74, 1.17) |

ACEi: Angiotensin converting enzyme inhibitor; ARB: Angiotensin receptor blocker; AKI-D: Acute kidney injury treated with dialysis; RR: Relative Risk; CI: Confidence Interval;

**RESULTS**

**Appendix G – Expanded Table 2 (Association between preoperative ACEi/ARB use and outcomes) showing all the variables considered in the logistic regression analysis**

| **Outcome: All-cause mortality** |  |
| --- | --- |
| **Variables** | **RR (95% CI)** |
|  |  |
| ACEi or ARB (Yes vs No) | 0.91 (0.87, 0.95) |
| Age | 1.05 (1.05, 1.06) |
| Sex (Female vs Male) | 0.82 (0.78, 0.85) |
| Chronic obstructive pulmonary disease | 1.62 (1.51, 1.73) |
| Cerebrovascular disease | 1.20 (1.14, 1.27) |
| Peripheral vascular disease | 1.25 (1.15, 1.36) |
| Coronary artery disease | 0.96 (0.91, 1.00) |
| Congestive heart failure | 1.57 (1.49, 1.65) |
| Chronic kidney disease | 1.65 (1.54, 1.77) |
| Chronic lung disease | 1.94 (1.53, 2.47) |
| Antidiabetic medication | 1.32 (1.26, 1.39) |
| β-blocker | 0.99 (0.94, 1.03) |
| Calcium channel blocker | 0.97 (0.93, 1.01) |
| Diuretic | 1.21 (1.15, 1.26) |
| Statin | 0.68 (0.65, 0.72) |
| Surgical Category |  |
| Cardiac vs Abdominal | 0.79 (0.75, 0.83) |
| Retroperitoneal vs Abdominal | 0.65 (0.60, 0.71) |
| Thoracic vs Abdominal | 1.85 (1.73, 1.97) |
| Vascular vs Abdominal | 0.92 (0.86, 0.98) |
| Era of surgery |  |
| 1999-2001 vs 1995-1998 | 0.99 (0.94, 1.05) |
| 2002-2004 vs 1995-1998 | 1.08 (1.02, 1.15) |
| 2005-2007 vs 1995-1998 | 1.06 (0.99, 1.13) |
| 2008-2010 vs 1995-1998 | 1.03 (0.97, 1.10) |

ACEi: Angiotensin converting enzyme inhibitor; ARB: Angiotensin receptor blocker; RR: Relative Risk; CI: Confidence Interval;

REFERENCES

1. von EE, Altman DG, Egger M, Pocock SJ, Gotzsche PC, Vandenbroucke JP: The Strengthening the Reporting of Observational Studies in Epidemiology (STROBE) statement: guidelines for reporting observational studies. *Lancet* 370:1453-1457, 2007

2. Juurlink DN, Preyra C, Croxford R, Chong A, Austin PC, Tu JV, Laupacis A: Canadian Institute for Health Information Discharge Abstract Database: A Validation Study. *ICES Investigative Report* 2006

3. Waikar SS, Wald R, Chertow GM, Curhan GC, Winkelmayer WC, Liangos O, Sosa MA, Jaber BL: Validity of International Classification of Diseases, Ninth Revision, Clinical Modification Codes for Acute Renal Failure. *J Am Soc Nephrol* 17:1688-1694, 2006

4. Ronksley PE, Tonelli M, Quan H, Manns BJ, James MT, Clement FM, Samuel S, Quinn RR, Ravani P, Brar SS, Hemmelgarn BR: Validating a case definition for chronic kidney disease using administrative data. *Nephrol Dial Transplant* 2011

5. Gershon AS, Wang C, Wilton AS, Raut R, To T: Trends in chronic obstructive pulmonary disease prevalence, incidence, and mortality in ontario, Canada, 1996 to 2007: a population-based study. *Arch Intern Med* 170:560-565, 2010

6. Liu L, Reeder B, Shuaib A, Mazagri R: Validity of stroke diagnosis on hospital discharge records in Saskatchewan, Canada: implications for stroke surveillance. *Cerebrovasc Dis* 9:224-230, 1999

7. Henderson T, Shepheard J, Sundararajan V: Quality of diagnosis and procedure coding in ICD-10 administrative data. *Med Care* 44:1011-1019, 2006

8. Thygesen SK, Christiansen CF, Christensen S, Lash TL, Sorensen HT: The predictive value of ICD-10 diagnostic coding used to assess Charlson comorbidity index conditions in the population-based Danish National Registry of Patients. *BMC Med Res Methodol* 11:83, 2011

9. Austin PC, Daly PA, Tu JV: A multicenter study of the coding accuracy of hospital discharge administrative data for patients admitted to cardiac care units in Ontario. *Am Heart J* 144:290-296, 2002

10. Kramer JR, Davila JA, Miller ED, Richardson P, Giordano TP, El-Serag HB: The validity of viral hepatitis and chronic liver disease diagnoses in Veterans Affairs administrative databases. *Aliment Pharmacol Ther* 27:274-282, 2008
